# Dietary Mitophagy Enhancer: A Strategy for Healthy Brain Aging?

**DOI:** 10.3390/antiox9100932

**Published:** 2020-09-29

**Authors:** Nimmy Varghese, Selina Werner, Amandine Grimm, Anne Eckert

**Affiliations:** 1 Neurobiology Lab for Brain Aging and Mental Health, Transfaculty Research Platform, Molecular & Cognitive Neuroscience, University of Basel, 4002 Basel, Switzerland; nimmy.varghese@unibas.ch (N.V.); selina.werner@unibas.ch (S.W.); amandine.grimm@unibas.ch (A.G.); 2 Psychiatric University Clinics Basel, Medical Faculty, University of Basel, 4002 Basel, Switzerland

**Keywords:** mitophagy, brain aging, antioxidants, diet, curcumin, spermidine, astaxanthin, resveratrol, hydroxytyrosol, oleuropein

## Abstract

Recently, nutritional interventions have received attention as promising approaches to promote human health during a lifespan. The Mediterranean and Okinawan diets have been associated with longevity and decreasing risk for age-related diseases in contrast to the Western diet. The effect might be due to several antioxidative bioactive compounds highly consumed in both diets, namely, resveratrol, hydroxytyrosol, oleuropein, curcumin, and spermidine. This review aims to address the underlying mechanisms of these compounds to enhance mental fitness throughout life with a focus on brain mitophagy. Mitophagy is the autophagic clearance of dysfunctional, redundant, and aged mitochondria. In aging and neurodegenerative disorders, mitophagy is crucial to preserve the autophagy mechanism of the whole cell, especially during oxidative stress. Growing evidence indicates that curcumin, astaxanthin, resveratrol, hydroxytyrosol, oleuropein, and spermidine might exert protective functions via antioxidative properties and as well the enhanced induction of mitophagy mediators. The compounds seem to upregulate mitophagy and thereby alleviate the clearance of dysfunctional and aged mitochondria as well as mitogenesis. Thus, the Mediterranean or Okinawan diet could represent a feasible nutritional approach to reduce the risk of developing age-related cognitive impairment and corresponding disorders via the stimulation of mitophagy and thereby ensure a balanced redox state of brain cells.

## 1. Introduction

**Figure 1:**
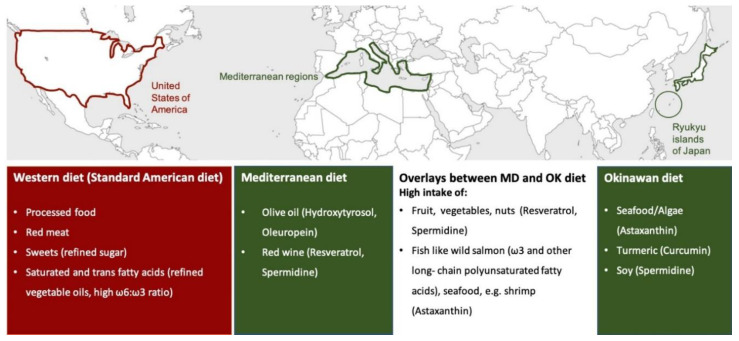
Characteristic bioactive compounds of the Mediterranean and Asian/Okinawan diet. The figure shows the geographic origin of three different types of diets: the Western, the Mediterranean, and the Asian (Okinawan) diet. The Mediterranean diet is defined as a dietary pattern of people living in countries that border the Mediterranean Sea, whereas the Okinawan diet originates from the eating habits of the indigenous people of the Japanese Ryukyu Islands (main island Okinawa). The Western diet is prevalent in developed countries, especially in the USA. Typical of the Mediterranean diet is the consumption of red wine with its active ingredients resveratrol and spermidine, and the intake of olive oil with its active ingredients oleuropein and hydroxytyrosol. All four compounds are associated with beneficial effects on human health. Characteristic food items of the Okinawan diet that are also known for their health benefits are soy products such as tofu (spermidine), turmeric (curcumin), seafood, and algae (astaxanthin). Similarities of both diets include the intake of a broad spectrum of antioxidants, polyphenols (like resveratrol), and spermidine from fruit and vegetable and the moderate-to-high consumption of seafood and fish (astaxanthin, healthy fatty acids). In contrast to both diets, the Western diet could have detrimental health consequences due to the high consumption of unhealthy fatty acids (saturated fatty acids), red meat, sweets, and highly processed food items. Abbreviations: MD = Mediterranean diet, OK = Okinawan diet, ω = omega, USA = United States of America.

The interest in nutritional interventions as a promising approach to counteract pathological brain aging and age-related neurodegenerative disorders has been rising in the last decade [1]. In this light, several bioactive compounds present in different foods have been found to be beneficial for human health during a lifespan [2]. This review focuses on two different diets, the Mediterranean diet and the Okinawan diet, which are both linked to longevity and decreasing risk for chronic age-related brain disorders (Figure 1) [3,4].

The Mediterranean diet, a traditional dietary pattern of the people living in the Mediterranean region (as shown in Figure 1), is known for its high consumption of olive oil, fruits, and vegetables, which are especially high in antioxidants, as well as moderate to high intake of fish, whole grain cereals, and red wine [1,5], whereas, the consumption of sweets, meat, and dairy products is rather low [6]. There are specific plant bioactives (e.g., resveratrol from red wine, hydroxytyrosol and oleuropein from olive oil) that are especially characteristic of the Mediterranean diet and its beneficial effects on human health (Figure 1) [6,7]. Initially, the Mediterranean diet became popular because of its historical lower rate of cardiovascular-related diseases in comparison to the Western diet, which is prevalent in developed countries, especially in the US [6]. Different from the Mediterranean diet and Asian diet, Western-type diets are dominated by convenience and highly processed foods such as processed and red meat, desserts, sweets, fried food, and a high intake of dairy products, containing a higher number of saturated fatty acids and refined grains, so that consumption of a Western diet could have detrimental consequences on health, due to the reduced intake of fresh fruit and vegetables in contrast to the excess consumption of saturated fatty acids, sugar, and sodium (Figure 1) [8]. Nowadays, several epidemiological studies, as reviewed by Casas and coworkers, have shown the potential of the Mediterranean diet as protection against a wide range of disease conditions, such as cancer, diabetes, atherosclerosis, cognitive disorders, and Alzheimer disease (AD) [9,10]. Since 2010, the Mediterranean diet is considered as a heritage by humanity by UNESCO by virtue of its beneficial properties [11]. As part of an Asian diet, the Okinawan diet has been found to be particularly interesting since the residents of the Japanese islands of Ryukyu (main island Okinawa) also represent one of the healthiest and longest-living people in the world (as seen in Figure 1) [3,12]. Their diet is characterized by a low caloric and fatty acid intake, high consumption of vegetables and soy products, and a moderate-to-high intake of fish and sea vegetables [3]. Le Couteur and coworkers named bitter melon, Okinawan tofu, turmeric, and seaweed as characteristics of the traditional Okinawan diet [12]. Similarities between the Mediterranean diet, as a European-based lifestyle, and the Okinawan diet, as part of an Asian-based lifestyle, are the high intake of antioxidants in the form of fruit and vegetables, the moderate-to-high consumption of fish, and the focus on healthy fats that are rich in omega 3 and lower in saturated fatty acids (Figure 1) [3]. In contrast to Asian diets, the Mediterranean diet is mostly abundant in isoflavones and curcumin [7]. Although further research is required, Pallauf and colleagues hypothesized that combining foods from both diets could improve the overall health of aging populations [7]. The removal of dysfunctional mitochondria called mitophagy is crucial for cell survival and health, especially for neurons, as impairments might generally happen with aging [13]. Recent studies have revealed abnormalities in mitochondrial function, including compromised clearing of dysfunctional mitochondria during brain aging and in the pathology of neurodegenerative disorders [14,15]. Therefore, as a next step, this review will target the underlying mechanisms of the most prominent bioactive compounds of both diets (namely, resveratrol from red wine, hydroxytyrosol and oleuropein from olive oil, astaxanthin from algae/seafood, curcumin from turmeric, as well as spermidine from soy products and vegetables) to promote health throughout life, with a specific focus on mitophagy and mitochondrial function in the brain.

## 2. Bioactive Compounds Promoting Health in the Mediterranean or Asian/Okinawan Diet

The Mediterranean diet and the Asian/Okinawan diet have both been linked to longevity and decreasing risk for chronic age-related diseases. Therefore, we selected the most prominent bioactive compounds of both diets to have a closer look at the biological properties that make them beneficial for human brain health during a lifespan. Due to the fact that turmeric and seaweed/seafood are traditional ingredients of the Okinawan diet, curcumin and astaxanthin were chosen as bioactive compounds; resveratrol (red wine) and hydroxytyrosol and oleuropein (olive oil) are well known in the Mediterranean diet (Figure 1). Although resveratrol is mostly associated with the Mediterranean diet, the highest natural concentration exists in Japanese knotweed, a spring vegetable from East Asia [16]. Hence, there might be an overlap of health-promoting bioactive compounds in the two types of diets, as astaxanthin can also be found in seafood (e.g., shrimp), which is as well consumed in Mediterranean areas. Spermidine is also present in both diets since it occurs in soy products and red wine but also in fresh fruit and vegetables, which are essential for the Mediterranean and Asian/Okinawan diets.

### 2.1. Curcumin (Turmeric)

Turmeric grows widely in different parts of India and Southeast Asia, as well as in tropical regions, and is well known as a spice in human diets [17]. Curcumin, a bright yellow-colored polyphenol that is derived from the rhizome of turmeric (Curcuma longa), is the main active compound of turmeric [18,19]. Turmeric or turmeric compounds like curcumin are used in traditional Indian medicine (Ayurvedic medicine) and other traditional medical systems for the treatment of a wide variety of diseases and conditions, such as pulmonary or gastrointestinal diseases [3,17]. Moreover, it is believed to possess a wide range of biological properties, including anti-inflammatory, antioxidant, as well as neuroprotective effects [17,18,19].

### 2.2. Astaxanthin (Algae, Seafood)

Astaxanthin is a xanthophyll carotenoid that is found in marine microorganisms, microalgae, fungi, yeast, and crustaceans like shrimp and lobster, as well as in reddish-colored fish like salmon [20,21]. Astaxanthin is considered to be one of the most potent antioxidants in nature and, as such, it is not only able to decrease oxidative stress in cell- and animal models but is also believed to have a positive effect on aging [21,22]. Besides its antioxidant effects, astaxanthin seems to exert anti-inflammatory, antitumor, and antiaging effects [20,23].

### 2.3. Resveratrol (Red Wine, Japanese Knotweed, Grapes)

Resveratrol is a natural stilbene polyphenolic compound that is prominent in red wine as well as in other food sources, including fruits like grapes, bananas, and blueberries, but also in spinach, peanuts, and cocoa [24,25,26]. Japanese knotweed possesses the highest naturally occurring levels of resveratrol [16]. Numerous biological properties of resveratrol have been reported, such as antioxidant, anti-inflammatory, antitumor, antiaging, and neuro- and cardioprotective effects [24,27,28].

### 2.4. Hydroxytyrosol, Oleuropein (Olive Oil)

Hydroxytyrosol and oleuropein are the major phenolic compounds found in olive oil and a major source of unsaturated fatty acids in the Mediterranean diet [29,30]. The relative amount of oleuropein and hydroxytyrosol in extra virgin olive can vary as it is influenced by different factors such as the variety and maturity of the olive fruits, the climate, and processing [11,31]. Hydroxytyrosol is a product of oleuropein hydrolysis that occurs during the maturation and storage of olive oil, as well by microbiota action in the organism after the consumption of olive oil [11,31]. Hydroxytyrosol can also be found in wine due to the ability of yeast to produce it during alcoholic fermentation [11]. Several in vitro and in vivo studies have shown the various health benefits of oleuropein and its derivates, including antioxidant, anti-inflammatory, antiviral, antitumor, cardioprotective, antidiabetic, and neuroprotective effects [31].

### 2.5. Spermidine (Fruit, Vegetable, Soybean)

Spermidine is a polyamine which exists in all living cells, including microorganism, plants, and animals [32]. There are three different sources for spermidine in humans: endogenous biosynthesis, exogenous food intake, and microbial activity in the intestines [33,34]. Additional supplementation of polyamine precursors, such as ornithine and related amino acids, is supposed to enhance polyamine synthesis [35]. Spermidine-rich sources are, on the one hand, unprocessed plant-derived foods, e.g., fresh green pepper, cauliflower, broccoli, nuts, wheat germ, and mushrooms and, on the other hand, products deriving from fermentation processes such as soybean, cheese, and red wine [36,37]. According to Madeo and colleagues, the highest amount of spermidine is found in the Mediterranean diet [38]. Spermidine exerts antioxidant, anti-inflammatory, and cardioprotective actions and is furthermore believed to preserve high-order brain functions [39]. Both Jing and coworkers and Madeo and coworkers have mentioned the potential of exogenous spermidine supply in prolonging the life span of several model organisms, including yeast, nematodes, and flies [40,41]. Jing and group postulate that spermidine might have an antiaging effect due to its autophagy-enhancing properties [40].

## 3. Autophagy and Mitophagy as a Crucial Cellular Mechanism Promoting Brain Health

In the interest of this review, it is necessary to comprehend the underlying effects of the compounds on the autophagy and mitophagy mechanism, especially in the brain. Autophagy is an essential recycling and eliminating mechanism targeting redundant, dysfunctional, and aged cellular components by alleviating lysosomal-mediated degradation [42,43]. In particular, the degradation of proteins, organelles, and cytosol occurs through their encapsulation by a double-membrane vesicle named autophagosome, followed by fusion with lysosomes [43,44]. Autophagy is essential because of its role in nutrient deprivation, cellular homeostasis, cell differentiation, and intracellular quality control [44,45]. The autophagy mechanism can be subdivided into three groups: chaperone-mediated autophagy, microautophagy, and macroautophagy [43,45,46].

(i) Chaperone-mediated autophagy is a chaperone-dependent selection, whereby specific consensus sequences containing proteins are detected and translocated to the lysosome via chaperone complexes.
(ii) Microautophagy is defined as the direct invagination of lipids, protein, or organelles by the lysosomal membrane, mediated by acidic hydrolases.
(iii) Macroautophagy is an extensively studied subgroup. Based on selection specificity, macroautophagy is further subdivided into (1) nonselective macroautophagy, characterized by the random destruction of cytoplasmic material and (2) selective macroautophagy, the selective clearance of specific organelles including peroxisomes (pexophagy), ER (ER-phagy or reticulophagy), and mitochondria (mitophagy) [45,47].

Macroautophagy plays a crucial role in intracellular quality control by only clearing out dysfunctional organelles and thereby protecting the whole cell from death. This review focuses on the positive influence of different compounds present in the Mediterranean and Asian/Okinawan diets on macroautophagy, especially mitophagy, in correlation to brain aging and Alzheimer’s disease. The mitophagy process is presented in more detail in the following chapter.

### 3.1. Mitophagy

Mitophagy is considered as the autophagic clearance of dysfunctional, redundant, and aged mitochondria [48]. The removal of dysfunctional mitochondria is an essential feature in the cell, based on the fundamental property of the mitochondria. Moreover, the role of mitophagy in aging and neurodegenerative disorders is crucial to preserving the autophagy mechanism of the whole cell, especially during oxidative stress in neurons [44,48]. In particular, to comprehend the importance of mitophagy in the cell, it is necessary to understand the function that the mitochondrion fulfills, especially in neurons.

#### 3.1.1. The General Concept of Mitochondria

Mitochondria, referred to as the “powerhouses of the cell”, are the primary energy providers of the cell [49,50]. The mitochondrial bioenergetics produce energy in the form of adenosine triphosphate (ATP) by oxidation of sugars, fats, and proteins via aerobic respiration [51]. The energy produced by the mitochondria is provided via the tricarboxylic acid (TCA) cycle, fatty acid oxidation, and oxidative phosphorylation (OXPHOS) pathways [2,51,52,53]. The number of mitochondria in different cell types varies depending on the energy demand. The brain is a high energy consumer, utilizing 20% of the body basal oxygen to maintain its property. For this reason, neuronal cells are highly dependent on the aerobic energy produced by the mitochondria; consistently, the number of mitochondria can reach up to two million in one neuron [54,55,56]. Besides the role of an energy factory in the neuron, mitochondria act on the regulation of intracellular calcium homeostasis, cell survival and function, synaptic plasticity, and neurotransmitter synthesis [52,57]. Mitochondria can be seen as paradoxical organelles. While mitochondria fulfill their function to generate energy, they produce harmful reactive oxygen species (ROS) and reactive nitrogen species (RNS), targeting themselves [58,59,60,61]. The oxidants produced by the mitochondria lead to mitochondrial malfunction [47,52,62]. Mitochondria possess their own DNA, called mitochondrial DNA (mtDNA). The mtDNA is localized in the mitochondrial matrix and encodes for 37 genes, including 13 genes crucial for mitochondrial respiration. Due to its localization in an oxidative environment, mtDNA is exposed to a high level of oxidative stress, leading to random mtDNA mutations that may impair mitochondrial respiration [51,63,64,65]. In order to target mtDNA, ROS and RNS can directly impair mitochondrial properties by damaging mitochondrial proteins and lipids, covering insufficient ATP production, an imbalance in cytoplasmic calcium buffering, and the enhanced release of proapoptotic factors. In a normal healthy condition, the quality control of mitochondria is counteracting the impairment, whereby mitochondrial dynamics play a crucial aspect, especially in the long-lived postmitotic neurons that are not replaced during life, which are normally are as old as the individual. Neuronal mitochondria, therefore, need constant rejuvenation [2,66,67].

#### 3.1.2. Mitochondrial Dynamics and Mitophagy

**Figure 2:**
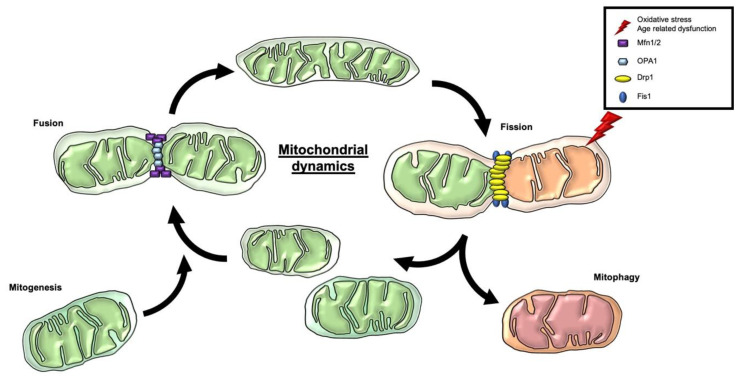
Mitochondrial dynamics, mitogenesis, and mitophagy as mitochondrial quality control. Mitochondria are dynamical organelles undergoing morphological changes by fusion or division events, named fusion and fission. OPA1 regulates the fusion of the inner IMM, while the fusion of the OMM is controlled by the two GTPase proteins Mfn 1/2 with these connecting adjoined mitochondria. Two dynamin-regulated GTPases are mainly involved in the event of fission, Drp1 and Fis1. Drp1 is recruited by Fis1 to the OMM. The oligomerization of Drp1 induces constriction and the mitochondrial cleavage. The mitochondrial dynamics are essential parameters of mitochondrial quality control, whereby the generation and clearance of mitochondria are essential contributors. Fission provides an initial step for the clearance of dysfunctional and aged mitochondria via mitophagy. The opposite mechanism of mitochondrial degradation is the generation of new mitochondria, named mitogenesis, and can deliver new mitochondria to the fusion mechanism. Abbreviations: IMM = inner mitochondrial membrane; OPA1 = optic atrophy 1; OMM = outer mitochondrial membrane; Mfn 1/2 = mitofusin 1 and 2; Drp1 = dynamin-related protein 1; Fis1 = fission protein 1; mitogenesis = mitochondrial biogenesis.

Mitochondria are dynamical organelles undergoing morphological changes by fusion or division in the cell. The dynamic network determines the position, shape, and size of the mitochondria in the cell [68,69]. The variety of shapes can vary from an interconnected tubular state to individual fragmented mitochondria. The state of the mitochondrial network affects function, maintenance, and distribution [70]. The mitochondrial morphology is defined by the balance between two opposite processes, named fusion and fission (Figure 2) [69].

The fusion mechanism is the union of mitochondria to form a tubular state. The major steps are the fusion of the two mitochondrial membranes at two independent events. The fusion of the inner mitochondrial membrane (IMM) is regulated by optic atrophy 1 (OPA1), while the fusion of the outer mitochondrial membrane (OMM) is controlled by the two GTPase proteins mitofusin (Mfn) 1 and 2 [51,71,72] with these connecting adjoined mitochondria [73]. Fusion allows the mitochondria to mix and to exchange mitochondrial content for maintaining a homogenous state. The interaction prevents the enrichment of damaged mtDNA by diluting the mutation [51,52,68,74]. Besides the beneficial dilution, fusion can have a higher affinity to spread infection as a side effect, contributing to an impairment of the overall quality control [45]. The fragmentation of the mitochondria characterizes fission. A heterogenous organelle population with a nonuniform distribution of the mtDNA is provided by fission [51]. Fission provides an initial step for the removal, redistribution, and degradation of mitochondria [52]. Two dynamin-regulated GTPases are mainly involved in the event, dynamin-related protein 1 (Drp1) and fission protein 1 (Fis1). Fis1 recruits Drp1 to the OMM. The oligomerization of Drp1 induces constriction and the mitochondrial cleavage [52,73]. Notably, the mitochondrial morphology seems to regulate the bioenergetics and vice versa [51,75]. The tubular state of the mitochondria is characterized by a higher bioenergetic efficiency than fission [68]. By environmental changes or stress, the mitochondrial morphology can differ depending on the energy request [69,70,74]. The mitochondrial dynamics are essential parameters of mitochondrial quality control, whereby the generation and clearance of mitochondria are essential contributors. The opposite mechanism of mitochondrial degradation is the generation of new mitochondria, named mitochondrial biogenesis (mitogenesis) [2]. The process is mainly mediated by the transcriptional coactivator peroxisome proliferator activated receptor gamma coactivator-1 alpha and beta (PGC-1 α/β) by inducing the transcription of nuclear respiratory factor (NRF) 1 and 2. In turn, NRF activates mitochondrial transcription factor A (TFAM), driving mitochondrial biogenesis [68,76]. Upstream regulators of PGC-1 α are the deacetylase silent mating type information regulation 2 homolog (SIRT) 1 and 3 and the transcription factors transcription factor EB (TFEB), cAMP response element-binding protein (CREB), and the forkhead transcription factor (FOXO1) [77,78,79,80]. AMP-activated protein kinase (AMPK) is one of the central upstream regulators of mitophagy and mitogenesis. As an energy sensor of the cells, it is essential to innate mitophagy and biogenesis [76]. Through oxidative-stress, induced mtDNA mutation activates the AMPK pathway and further inhibits the mammalian target of rapamycin (mTOR) [46]. AMPK, in turn, stimulates mitophagy via Unc-51-like kinase 1 (ULK1) and mitogenesis via SIRT1 and PGC-1 α pathways. On the other hand, mTOR blocks mitophagy by inhibiting ULK1 [2,43,47,81,82], as well as downstream signaling of ribosomal protein S6 kinase beta-1 (S6K1). In turn, S6K1 also suppresses mitophagy by indirectly inhibiting ULK1 and Beclin-1 [83,84]. The upstream modulator of mTOR is the insulin receptor/insulin-like growth factor receptor (IR/IGF-R), which activates phosphoinositide 3-kinases (PI3K), followed by the activation of protein kinase B (AKT), which in turn activates mTOR [80,83,84]. Extracellular signal-regulated kinase (ERK), in turn, phosphorylates Drp1 for mitochondrial fragmentation and stimulates mitophagy due to the activation of IR/IGF-R signals [85]. Paradoxically, the mitogenesis promoter PGC-1 α also interferes with mitophagy by regulating lysosomal biogenesis via TFEB, thereby representing a strong correlation between mitogenesis and mitophagy [77,78]. AMPK also targets FOXO1 and -3 [84]. FOXO, in turn, increases the transcription of essential mitophagy mediators and proteins [86].

#### 3.1.3. Subtypes of Mitophagy

Mitophagy can be separated into three subgroups depending on how they are marked for clearance: (i) cardiolipin-mediated mitophagy, (ii) receptor-mediated mitophagy, and (iii) ubiquitin-mediated mitophagy [87].

**Figure 3:**
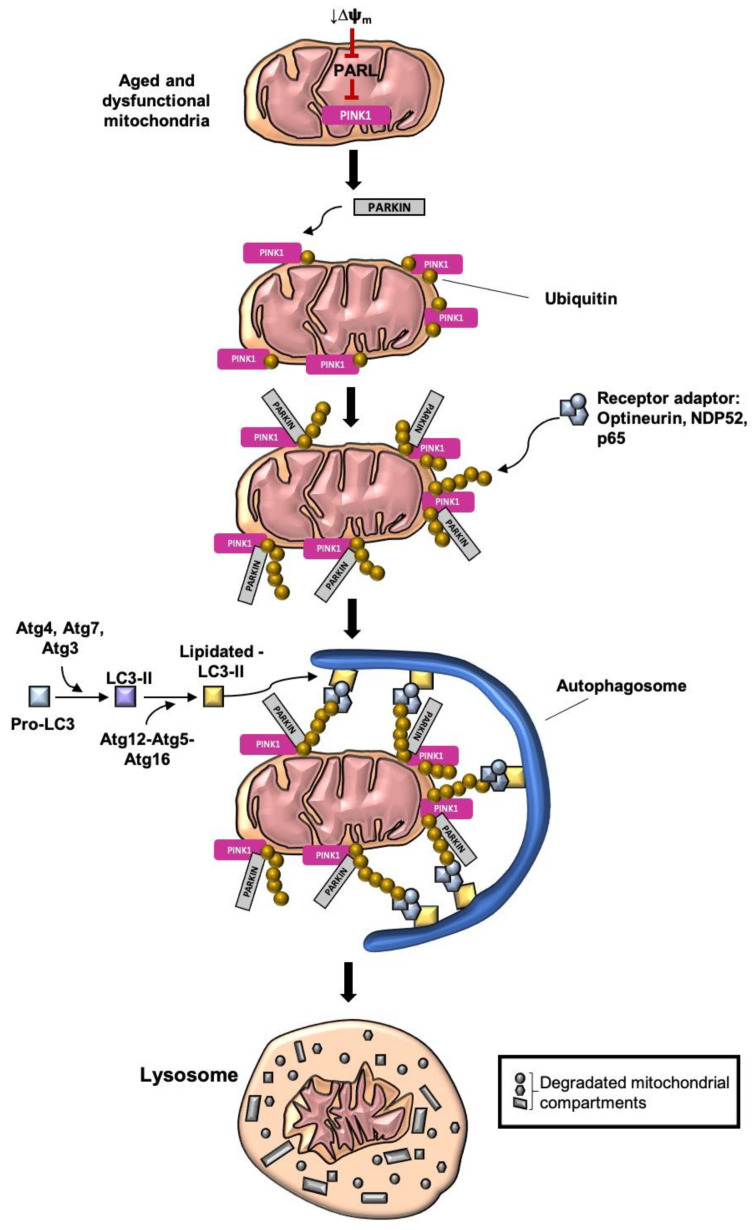
Ubiquitin-mediated mitophagy—the PINK1–PARKIN-mediated pathway. One of the prominent members of ubiquitin-mediated mitophagy is the PINK1–PARKIN pathway. The loss of the MMP is considered to be the primary initiator of mitophagy. The decline of MMP promotes the accumulation of PINK1 on the OMM by destabilizing the IMM protease, PARL. PINK1 fulfills the function of posttranslationally labeling OMM proteins with ubiquitin and recruits and activates the cytosolic E3 ubiquitin ligase PARKIN. The function of PARKIN is to ubiquitinate OMM proteins, such as Mfn1/2, VDAC, and TOM proteins, triggering the formation of ubiquitin chains. The polyubiquitin chains mark the mitochondrion for removal by targeting the receptors such as optineurin, NDP52, and phosphorylated p65. These adaptor receptors are bound by bifunctional adapter proteins, consisting of ubiquitin-binding motifs and LIR alleviating the binding to Atg8-like proteins such as the LC3-II. LC3-II is generated by converting pro-LC3 in LC3-I and further to LC3-II (PE-conjugated LC3) by the protease activity of Atg4, Atg7, and Atg3. In the next step, LC3-II is lipidated by the E3-like active complex Atg12–Atg5–Atg16. The lipid-conjugated LC3 facilitates the attachment to the phagophore membrane and furthers the full engulfment of the mitochondria by forming an autophagosome. In the last step, the autophagosome fuses with the lysosome, whereby the internal microenvironment of the lysosome turns the internal environment acidic. Then, acidic lysosomal enzymes are activated, leading to the degradation of the mitochondrion. The final content is then disposed of or recycled to generate new mitochondria. Abbreviation: MMP/ΔΨm = mitochondrial membrane potential; PINK1 = phosphatase and tensin homolog PTEN-induced putative protein kinase 1; PARL = presenilin-associated rhomboid-like; Mfn1/2 = mitofusin; VDAC = voltage-dependent anion channel 1; TOM = translocase of the outer membrane; NDP52 = 52 kDa nuclear dot protein; LIR = LC3-interacting regions; Atg = autophagy-related gene; LC3-II = microtubule-associated protein 1A/1B-light chain 3.

(i) Cardiolipin-mediated mitophagy is a recently discovered mitophagy pathway. In this pathway, lipids localized on the OMM contribute to directly lure the mitophagy machinery. As a result of this, a dimeric phospholipid, cardiolipin, is anchored to the OMM of a dysfunctional mitochondrion. On the OMM, cardiolipin connects directly with LC3-II (lipidated) [78,87].
(ii) In receptor-mediated mitophagy, the membrane cargo-receptors are directly activated and interact with autophagosome marker proteins (LC3/Atg8-like). The facilitation of the receptor-mediated pathway is provided by receptors such as Nip3-like protein X (NIX), Bscl2 interaction protein 3 (BNIP3), FUN14 domain containing 1 (FUNDC1), and AMBRA1 and SMAD ubiquitination regulatory factor 1 (SMURF1), FK 506 binding protein 8 (FKBP8) and prohibitin 2 (PHB2) [45]. Hypoxia-inducible factor α (HIF-1 α) and FOXO are some of the upstream stimulators of these cargo-receptors, which facilitate the receptor-mediated mitophagy pathway [77,80,86].
(iii) Ubiquitin-mediated mitophagy is induced by a massive ubiquitination of mitochondrial OMM proteins [45]. One of the prominent members of ubiquitin-mediated mitophagy is the PINK1–PARKIN pathway. The PINK1–PARKIN pathway is the best-known pathway in the correlation of mitophagy and neurodegeneration (Figure 3) [87,88].

The loss of mitochondrial membrane potential (MMP; ΔΨm) is considered to be the primary initiator of mitophagy [52,68,89]. MMP is the main driving force of Complex V, the ATPase. The production of ATP from ATPase is the last step of OXPHOS [51,90,91]. Notably, the fragmentation of the mitochondrion ensures efficient engulfment by the phagosome by isolating the dysfunctional mitochondrion from the network [45,51,57,68,92], whereas increased activation of fusion protects the mitochondria from degradation [88]. The decline of MMP promotes the accumulation of phosphatase and tensin homolog [42] PTEN-induced putative protein kinase 1 (PINK1) on the OMM [52]. Under basal conditions, PINK1 is translocated via the translocase of the outer membrane (TOM) and the translocase of the inner membrane (TIM) complex to the IMM, where it is destabilized by the IMM protease presenilin-associated rhomboid-like (PARL). The loss of MMP inhibits PINK1’s translocation to the IMM by inducing the degradation of PARL [45,46,47,88]. PINK1, as a mitophagy inducer, fulfills the function of posttranslationally labeling OMM proteins with ubiquitin and recruits and activates the cytosolic E_3_ ubiquitin ligase PARKIN. The function of PARKIN has been shown to ubiquitinate OMM proteins, such as mitofusin (Mfn1/2), voltage-dependent anion channel 1 (VDAC), and TOM proteins, triggering the formation of ubiquitin chains [45,46,88]. The polyubiquitin chains mark the mitochondrion for the removal by targeting the receptors such as optineurin, 52-kDa nuclear dot protein (NDP_52_), and phosphorylated p65 [42,48,87]. These adaptor receptors are bound by bifunctional adapter proteins consisting of ubiquitin-binding motifs and LC3-interacting regions (LIR), alleviating the binding to Atg8-like proteins such as the microtubule-associated protein 1A/1B-light chain 3 (LC3)-II [45]. The generation of LC3-II is done by converting pro-LC3 to LC3-I and further to LC3-II (PE-conjugated LC3) by the protease activity of Atg4, Atg7, and Atg3. In the next step, LC3-II is lipidated by the E3-like active complex Atg12–Atg5–Atg16. The lipid-conjugated LC3 facilitates the attachment to the phagophore membrane and furthers the full engulfment of the mitochondria by forming an autophagosome [42,46,48,52,73,87,93]. The upstream-activator of the Atg12–Atg5–Atg16 complex is ULK1 via the PI3K complex [80,84]. Another function of ULK1 is to activate Beclin-1, which also alleviates the recruitment of PARKIN on the OMM. Interestingly, the involvement of PINK1 is not essential. In the absence of PINK1, PARKIN can still be recruited to the mitochondrial membrane [80]. Lastly, the autophagosome fuses with the lysosome, whereby the internal microenvironment of the lysosome turns the internal environment acidic. Then, acidic lysosomal enzymes are activated, leading to degradation of the mitochondrion. The final content is then disposed of or recycled [62].

### 3.2. The Physiological Role of Mitophagy in Response to Oxidative Stress

Under basal conditions, mitophagy plays a beneficial role in regulating the number of mitochondria depending on energy demand [48]. The mitophagy process degrades or recycles dysfunctional mitochondria to sustain the energy homeostasis and maintain quality control. Importantly, mitophagy has to fulfill these functions during various stress events, including metabolic and bioenergetic stress and mitochondrial damage [42,47,48,94]. A prominent trigger of mitophagy is oxidative stress in the form of ROS and RNS, impairing the mitochondria properties. The decisive impairment is the decline of ATP production linked to a loss of MMP, leading to the activation of mitophagy [46,94]. The two processes, mitophagy and biogenesis, define the mitochondrial content in the cell by providing quality control of the mitochondria. In a healthy cell, a balance between mitochondrial degradation, biogenesis, and dynamics is maintained [68,95,96]. The mitophagy pathway intervenes in the process of whole-cell degradation, but it only phagocytoses the mitochondria [13]. The selective removal is necessary for maintaining neuronal homeostasis [97].

### 3.3. The Emerging Role of Mitophagy in the Aging Brain and Age-Related Neurodegenerative Disorders—Alzheimer’s Disease

In the aging brain, the mitochondrial dynamics, mitogenesis, and mitophagy were shown to be altered. The biogenesis and degradation mechanism in age declines, leading to the accumulation of damaged and mutated mitochondria. Oxidative stress is considered one of the primary mediators of mitochondrial dysfunction and decline in mitophagy in aging. With age, the overproduction of ROS is mainly caused by a decline in the antioxidant defense and the loss of the clearance of dysfunctional mitochondria [52,98]. As a consequence, the mitochondrial DNA (mtDNA) is exposed to a highly toxic environment, leading to the accumulation of mutations [52,99]. From the aspect of redox biology, on the one hand, oxidative stress triggers mitophagy, but on the other hand, excessive targeting of oxidative stress leads to a dysfunction of mitophagy and inhibition of the process [46]. Due to their peerless properties as postmitotic-differentiated cells, neurons are strongly dependent on the controlled clearance of impaired proteins and organelles. Efficient removal of aggregated proteins during the aging of the neurons and dysfunctional or aged organelles is essential to prevent elevated cellular stress and neurodegenerative mechanisms [97,100]. A decline in mitophagy impacts mitostasis and may lead to a deficient function of the postmitotic neuron and further to cell death [42]. AD is the progressive, irreversible, and most common neurodegenerative disorder represented by cognitive defects, memory loss, and neuronal death [52]. The pathology of AD is characterized by the accumulation of intracellular hyperphosphorylated tau (p-tau) and extracellular amyloid ß-peptide (Aß). The accumulation of these proteins impairs the mitochondrial property, resulting in impairment in OXPHOS, ROS production, and mitochondrial dynamics and further to neuronal toxicity and death [42,47,66,100,101,102]. Regarding neurodegenerative disorders, the interest in mitophagy pathways has risen in the last decades, particularly on the PINK1-PARKIN pathway [42,87,100]. In AD, the reduction of PINK1 and PARKIN proteins leads to insufficient activation of the clearance of the mitochondria. It results in the accumulation of dysfunctional mitochondria and further to the rise of hyperphosphorylation of tau and synaptic dysfunction [43]

## 4. Mitophagy-Related Mechanism of Bioactive Compounds in the Mediterranean and Asian/Okinawan Diets

In this section of the review, the underlying targets of the bioactive food compounds regarding mitochondrial dynamics and mitogenesis, with a special focus on mitophagy in the brain, are highlighted in Table 1.

### 4.1. Summary of the Mitophagy-Related Mechanism of Bioactive Compounds of the Mediterranean and Asian/Okinawan Diets

**Figure 4:**
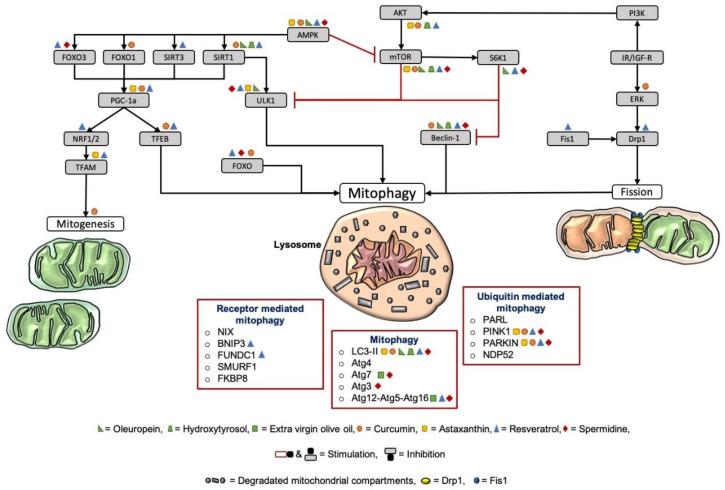
Target points of the dietary compounds on mitochondrial dynamics and mitogenesis, with a particular focus on mitophagy. AMPK is one of the central upstream regulators of mitophagy and mitogenesis. Through oxidative-stress, induced mtDNA mutation activates the AMPK pathway and further inhibits the mTOR [46]. AMPK, in turn, stimulates mitophagy via ULK1 and mitogenesis via the PGC-1 α pathway. The main upstream mediators of PGC-1 α are FOXO1/3 and SIRT1/3. PGC-1 α, in turn, activates mitophagy via NRF1/2 and TFAM. Paradoxically, the mitogenesis promoter PGC-1 α also interferes with mitophagy by regulating lysosomal biogenesis via TFEB. Further, AMPK targets FOXO1 and -3. FOXO, in turn, increases the transcription of essential mitophagy mediators and proteins. On the other hand, mTOR blocks mitophagy by inhibiting ULK1 as well as downstream signaling of ribosomal protein S6K1, which, in turn, also suppresses mitophagy by indirectly inhibiting ULK1 and Beclin-1. The upstream modulator of mTOR is the IR/IGF-R, which activates PI3K, followed by activation of AKT, which activates mTOR. ERK, in turn, phosphorylates Drp1 for mitochondrial fragmentation and stimulates mitophagy due to the activation of IR/IGF-R signals. In general, Fis1 is recruiting Drp1 to the OMM to induce mitochondrial fission. In this figure, the target points of the most prominent compounds of both the Mediterranean and the Asian/Okinawan diets are displayed. Hydroxytyrosol and oleuropein from olive oil, curcumin, astaxanthin, resveratrol, and spermidine were all found to stimulate mitochondrial dynamics, mitogenesis, and, especially, mitophagy. Abbreviation: AMPK = AMP-activated protein kinase; mtDNA = mitochondrial DNA; mTOR = mammalian target of rapamycin; ULK1 = unc-51-like kinase 1; PGC-1 α = peroxisome proliferator-activated receptor gamma coactivator 1-alpha; FOXO1/3 = forkhead transcription factor 1/3; SIRT1/3 = silent mating type information regulation 2 homolog 1/3; NRF1/2 = nuclear respiratory factor 1 and 2; TFAM = mitochondrial transcription factor A; TFEB = transcription factor EB; S6K1 = S6 kinase beta-1; IR/IGF-R = insulin receptor/insulin-like growth factor receptor; PI3K = phosphoinositide 3-kinase; AKT = protein kinase B; ERK = extracellular signal-regulated kinase; Drp1 = dynamin-related protein 1; Fis1 = fission protein 1; OMM = outer mitochondrial membrane.

The underlying targets of the bioactive food compounds regarding mitochondrial dynamics and mitogenesis, with a special focus on mitophagy, are visualized in Figure 4.

#### 4.1.1. Curcumin

As seen in Table 1, curcumin is supposed to exert protective functions via the induction of mitophagy. In detail, its effect on mitophagy might be due to its ability to inhibit the Akt/mTOR pathway, to enhance FOXO1, and to directly induce the PINK1–PARKIN pathway (Figure 4). Curcumin was also found to elevate mitochondrial biogenesis and genes related to mitochondrial biogenesis, such as SIRT1 and PGC-1α. Regarding Table 1, curcumin was able to induce mitophagy in the brain in U87-MG and U373-MG malignant glioma cells [104]. Wang and colleagues also suggest that curcumin is capable of exerting health benefits by improving mitophagy and restoring the mitochondrial function of neurons in rats after brain ischemia–reperfusion [145]. Furthermore, Liao and colleagues examined the effects of curcumin on aging in *Caenorhabditis elegans* (*C. elegans*) and showed that curcumin was able to prolong lifespan and decrease the level of ROS [18]. Moreover, dietary curcumin is able to reduce oxidative stress, improve redox status, and, therefore, lessens mitochondrial damage [108]. However, its therapeutic oral use for humans is still under debate due to its relatively low bioavailability. Currently, several strategies are being explored, with the aim to improve the absorption and solubility of curcumin, such as inhibiting the curcumin metabolism with adjuvants or novel liquid and solid oral delivery systems [146].

#### 4.1.2. Astaxanthin

Astaxanthin was shown to induce mitophagy through the activation of PINK1 and PARKIN and the inhibition of AKT/mTOR (Table 1). Moreover, astaxanthin was able to enhance mitogenesis via the generation of PGC-1α and TFAM (Figure 4). Yazaki and group reported that astaxanthin has not only strong antioxidant capacities but was also able to increase the lifespan of *C. elegans* [147]. The review of Kidd and colleagues highlights the effects of astaxanthin in aging and age-related disorders and further reports that astaxanthin was able to improve cognition in AD [148]. Unfortunately, studies investigating the underlying effects of astaxanthin on mitophagy in the brain are still missing.

#### 4.1.3. Resveratrol

As seen in Figure 4, resveratrol regulates cellular signaling pathways that induce mitophagy (e.g., FOXO3a, PINK1, PARKIN, FUNDC1, BNIP3, AKT/mTOR, and Drp1) and mitochondrial biogenesis (e.g., SIRT1, PGC-1α, and TFAM). The treatment with resveratrol is reported to maintain cellular homeostasis and improve antioxidant capacity [24]. Resveratrol is capable of upregulating mitophagy in the brain, as indicated in several in vivo studies. Wang and coworkers showed that resveratrol lessened the pathological damage in the frontal cortex and hippocampus in a rat model of chronic cerebral hypofusion via stimulating mitophagy [125]. Guo and group, as well as Chen and colleagues, mentioned the protective effects of resveratrol through activating mitophagy in the brains of rats [126,127]. Resveratrol was also found to promote mitophagy in neuronal cell cultures [130]. Similar to the other bioactive food compounds, resveratrol may promote organismal healthspan through autophagy enhancement [149]. Valenzano and coworkers’ findings are in line with this hypothesis, as food supplementation with resveratrol extended the lifespan and retarded the expression of age-related traits in the fish model *Nothobranchius furzeri* [150]. Further, resveratrol was found to counteract the age-related decrease in mitophagy in aging zebrafish (*Danio rerio*) [132].

#### 4.1.4. Olive Oil

Both Oleuropein and hydroxytyrosol are capable of enhancing mitophagy through the upregulation of mitophagy markers such as LC3-II, Beclin, SIRT1, and ULK1, among others, and the downregulation of S6K1 and AKT/mTOR (see Table 1, Figure 4). De Pablos and colleagues and Cetrullo and coworkers reported that the increase in mitophagy by hydroxytyrosol, a derivate of oleuropein, was furthermore linked to a decrease in oxidative stress, mitochondrial dysfunction, and cell death [11,30]. Notably, extra virgin olive oil administration to a triple-transgenic mice model of Alzheimer’s disease moreover ameliorated memory function, which was associated with autophagy activation [115]. Rigacci and coworkers highlighted the beneficial effects of oleuropein treatment regarding the stimulation of mitophagy in the brain in neuroblastoma cells and in a transgenic AD mice model [112]. Moreover, mice brain showed an increase in mitophagy markers after oleuropein feeding, which was confirmed in neuroblastoma cells [113].

#### 4.1.5. Spermidine

As seen in Table 1, spermidine induces mitophagy in animal and cell models through the upregulation of mitophagy-related markers such as Beclin-1, LC3-II, PINK1, PARKIN, ULK1, Atg, and AMPK and through the inhibition of mTOR. Spermidine was able to induce mitophagy in mouse neuroblastoma cells [40], as well as in the brain of aging mice models [141]. Several studies reported that spermidine consumption not only suppresses aging in yeast, flies, worms, human cells, and mice but also prolongs the lifespan of several model organisms and lessens oxidative stress in aging mice due to autophagy [151]. Sharma and group also support this hypothesis by stating that spermidine counteracts age-associated cell death by activating autophagy-related mechanisms and reducing the formation of reactive oxygen species [152].

## 5. Discussion

From a nutritional perspective, there is a significant overlap between the Mediterranean and the Asian diets, especially the Okinawan diet, despite the geographic distance and cultural differences. Both diets are characterized by a high intake of antioxidants in the form of fruit and vegetables, a moderate-to-high consumption of fish, and a focus on healthy fatty acids (rich in ω3, lower in saturated fats) [3]. Another similarity between these two diets is their believed benefits regarding healthy aging, the promotion of longevity, and the reduced risk for age-related diseases [3]. Apart from poor diet, smoking, obesity, and mental and physical inactivity are linked to an increased risk of AD and cognitive decline. As reviewed by Crous-Bou and coworkers, a third of AD cases are related to modifiable risk factors showing the potential of lifestyle interventions such as an adherence to the Mediterranean diet or physical exercise [153]. Several epidemiological studies point out the beneficial effects of the Mediterranean diet on cognitive impairment and AD risk. Since cognitive decline is an early sign of AD, preventive strategies such as diets to lower the role of modifiable risk factors in AD have become essential [154]. In this context, a study pointed out that the intake of a Mediterranean diet consistently increased the levels of cognitive function in elderly men and women over a period of eleven years [155]. In addition, adherence to the Mediterranean diet results not only in a reduction of cognitive decline and lower risk of AD development but also a decreased risk of mild cognitive impairment (MCI) and conversion from MCI to AD [156]. Moreover, the Mediterranean diet has been linked to a lower cognitive impairment in a population of 1410 Bordeaux citizens, as evaluated by the Mini-Mental State Examination [157]. Another study of 2258 New York City residents is also in line with this finding as high adherence to the Mediterranean diet decreased the risk of AD [158]. A study of 110 healthy elderly people, subdivided into 55 subjects receiving a Mediterranean diet and 55 subjects taking a Mediterranean diet plus extra virgin olive oil, shows that the effect on cognitive function was higher in people consuming a Mediterranean diet with additional supplementation of extra virgin olive oil [159]. Therefore, the consumption of bioactive food compounds, as present in olive oil itself, seems to have a neuroprotective effect and is moreover able to potentiate the beneficial properties of the Mediterranean diet. Berr and colleagues’ finding is in line with this hypothesis as they followed 6947 subjects and revealed that the intensive use of extra virgin olive oil attenuated cognitive decline compared to those who did not consume olive oil [160]. A clinical trial of resveratrol, a well-known compound of the Mediterranean diet, which also occurs in high concentrations in Japanese knotweed, reported less deterioration in the Mini-Mental State Examination and the Alzheimer’s Disease Assessment Scale on a cognitive subscale of 39 subjects with mild to moderate AD [161]. Although no epidemiological studies have shown the effect of the Okinawan diet on AD and cognitive impairment, there are studies regarding the beneficial effect of astaxanthin and curcumin, two bioactive compounds that are found in food items that are highly consumed in Okinawan cuisine. Astaxanthin supplementation was reported to improve cognitive function in 96 subjects with age-related forgetfulness [162] and to elevate psychomotor speed and processing speed in 21 people with mild cognitive impairment [163]. In 40 nondemented older subjects, curcumin improved memory and attention performance [164] and promoted the working memory and sustained attention in a clinical trial of 60 healthy elderly [165]. In the Okinawan diet, besides several beneficial food bioactives, caloric restriction seems to be an important contributor to the longevity phenotype [166]. Of note, similar to key compounds of the Okinawan diet, caloric restriction has been found to improve mitochondrial biogenesis and mitophagy in aging [2]. Spermidine occurs in both the Mediterranean and Okinawan diets, e.g., in red wine, fresh vegetables and fruit, soy products, nuts, and mushrooms. Despite the fact that clinical studies of spermidine are limited, spermidine supplementation was found to exert protective effects against age-induced memory impairment in *Drosophila melanogaster* via the recuperation of autophagy [167] and to improve memory performance in a phase IIa trial with 30 older adults with subjective cognitive decline [39]. This review addressed the underlying mechanisms of the most prominent bioactive compounds of the Mediterranean and Okinawan diets to promote mental fitness throughout life, with a focus on mitophagy. Accumulating evidence reveals that curcumin, astaxanthin, resveratrol, hydroxytyrosol, oleuropein, and spermidine exert their protective functions via the enhanced induction of mitophagy mediators. The compounds promote the upregulation of mitophagy and thereby alleviate the clearance of dysfunctional and aged mitochondria as well as the generation of new mitochondria. In addition, all of the mentioned bioactive food compounds also possess antioxidative properties, which seem to be necessary since oxidative stress is considered as one of the primary mediators of mitochondrial dysfunction and decline in mitophagy in aging and AD [13,168] (see Section 3.3). Our findings are in line with the meta-analysis of Cao and coworkers, reporting that the intake of a Mediterranean diet, antioxidants, and unsaturated fatty acids decreases the risk for dementia [169]. Nevertheless, the understanding of the protective effects of fish, vegetables, and fruit, which are naturally occurring sources of antioxidants, requires more research [169]. Although studies on brain mitophagy are limited, we speculate that the different compounds may also provide the same effect regarding the upregulation of mitophagy mediators on mitophagy in neurons. Neurons are dependent on mitochondrial respiration as the energy source and are crucially affected by mitochondrial dysfunction. The efficient removal of aggregated proteins and dysfunctional or aged organelles is therefore essential to prevent elevated cellular stress and further neurodegenerative mechanisms. The protective effect of free radical scavengers, antioxidants, and mitophagy enhancers seems to be the main mechanism of longevity and of decreasing the risk for neurodegenerative diseases [40].

## 6. Conclusions

One of the advantages of bioactive food compounds is the long history of their use in nutrition, indicating that they are well-tolerated. In this light, the Mediterranean or Okinawan diet could represent a feasible nutritional approach to reduce the risk of developing cognitive impairment and age-related neurodegenerative disorders like AD by stimulating mitophagy and ensuring a balanced redox state of brain cells. Moreover, we hypothesize that the combination of both diets or the prominent pharmacologically active compounds of the Mediterranean- and Asian-style diets might be even more beneficial due to the synergistic effects of the compounds. Further research is required, focusing on the most prominent pharmacologically active food compounds and further including them into the Western diet as a strategy to promote overall health in Western countries.

## Figures and Tables

**Table 1:** Mechanisms of bioactive food compounds in mitophagy.

Natural Compound	Dose (Duration)	Experimental Model	Principal Effect	Refs
**Curcumin**				
**In vitro**	5–20 µM (4 h)	EA.hy 926 human umbilical vein endothelial cells	↓AKT ↓mTOR	[103]
	40 μM (48 h)	U87-MG, U373-MG malignant glioma cells	↓AKT ↓mTOR ↓S6K1 ↑ERK1/2 ↑LC3-II	[104]
	10, 20, 40 μM (20 h)	HCT 116 human colon cancer cells	↑LC3-II	[105]
	1, 5, 10 μM (24 h)	HUVECs human umbilical vein endothelial cells from umbilical cords	↓AKT ↓mTOR ↑FOXO1 ↑Beclin-1 ↑LC3-II	[106]
	1–20 μM (6/8 d)	Adipocytes isolated from rats	↑AMPK ↑PGC-1α	[107]
	10 μM (12 h)	IPEC-J2 intestinal porcine epithelial cells	↑AMPK ↑TFEB ↑PINK1 ↑PARKIN	[108]
**In vivo**	200 mg/kg/d (14 d)	Piglets (jejunal tissue)	↑AMPK ↑TFEB ↑PINK1 ↑PARKIN	[108]
	50 mg/kg/d, 100 mg/kg/d (28 d)	Rats (skeletal muscle)	↑mitochondrial biogenesis ↑AMPK ↑PGC-1α ↑SIRT1	[109]
**Astaxanthin**				
**In vitro**	25, 50 nM (3 h)	AGS human gastric epithelial adenocarcinoma cells	↑AMPK ↓AKT ↓mTOR ↑ULK1 ↑LC3-II	[23]
**In vivo**	200 mg/kg/d (11 w)	Rats (primary aortic vascular smooth muscle cells VSMC)	↑PGC-1α ↑TFAM ↑PINK1 ↑PARKIN	[110]
**Oleuropein**				
**In vitro**	100 μM (6 h)	Neonatal rat cardiomyocytes	↑SIRT1 ↑Beclin-1 ↑LC3-II	[111]
	50 μM (4 h)	SH-SY5Y human neuroblastoma cells	↑AMPK	[112]
	9–100 µM (6 h)	N2a mouse neuroblastoma cells	↓S6K1 ↑Beclin-1	[113]
**In vivo**	50 mg/kg/d (8 w)	TgCRND8 transgenic Alzheimer’s disease mice model (hippocampal and cortical tissue)	↑Beclin-1 ↑LC3	[113]
	50 mg/kg/d (8 w)	TgCRND8 mice transgenic Alzheimer’s disease mice model (hippocampal and cortical tissue)	↑AMPK ↓mTOR ↓S6K1	[112]
	3% (16 w)	Mice (liver tissue)	↑ULK1 ↑Beclin-1 ↑LC3-II	[114]
**Extra virgin olive oil**				
**In vivo**	EVOO rich diet (6 m)	3xTg triple transgenic AD mice model (cortical tissue)	↑Atg5 ↑Atg7	[115]
**Hydroxytyrosol**				
**In vitro**	100 μM (30 min)	C-28/I2 human chondrocytes and primary osteoarthritis chondrocytes	↑SIRT1 ↑LC3-II	[30]
	12.5, 25, 50, 100, 200, 400 μM (24 h)	Vascular adventitial fibroblasts isolated from thoracic aorta of rats	↓AKT ↓mTOR ↑Beclin-1 ↑SIRT1 ↑LC3	[116]
**Resveratrol**				
**In vitro**	20 µM (4 d/16 d)	Granulosa cells and oocytes derived from aged cows	↑SIRT1 ↑LC3	[117]
	50 μM (2 h)	HeLa human cervical cancer cells	↓mTOR ↑ULK1 ↑LC3	[118]
	100 uM (6 h)	HCT 116 colon carcinoma cells	↑SIRT1 ↑LC3-II/LC3-I	[119]
	5, 10, 15 μM (2 d)	Human podocytes	↑Atg5 ↑LC3-II	[120]
	50 μmol (48 h)	HMrSV5 immortalized human peritoneal mesothelial cells	↑AMPK ↓mTOR ↑LC3-II	[121]
	10 µM (1 h)	HUVECs human umbilical vein endothelial cells	↑LC3-II/LC3-I	[122]
	0.1, 1 µM (1 h)	H9c2 cardiac myoblast cells	↑AMPK ↓mTOR ↑Beclin-1 ↑Atg5 ↑LC3-II	[123]
	50, 100 μM (12 h)	H9c2 cardiac myoblast cells	↑Drp1 ↑PINK1 ↑PARKIN ↑LC3-II	[124]
**In vivo**	50 mg/kg/d (9 w)	Rats (brain tissue)	↓AKT ↓mTOR ↓S6K1 ↑Beclin-1 ↑LC3-II	[125]
	10 mg/kg/d for 12 weeks	Db/db diabetic mice model (kidney tissue)	↑Atg5 ↑LC3-II	[120]
	60 mg/kg/d (24 h)	Rats (brain tissue)	↓AKT ↓mTOR ↑Beclin-1 ↑LC3-II	[126]
	30 mg/kg/d (21 d)	RSV respiratory syncytial virus rats (hippocampal tissue)	↑AMPK ↑PGC-1α ↑NRF1 ↑TFAM ↑Beclin-1 ↑PINK1 ↑PARKIN ↑LC3-II/LC3-I	[127]
	0.04, 0.4, 4 g/kg/d (56 w)	Mdx Duchenne muscular dystrophy mice model (quadriceps muscle tissue)	↑SIRT1 ↑Beclin-1 ↑TFEB ↑PINK-1 ↑PARKIN ↑BNIP3 ↑FUNDC1 ↑Atg5 ↑LC3-II/LC3-I	[128]
	5, 50, 500 mg/kg/d (65 w)	Mdx Duchenne muscular dystrophy mice model (cardiac tissue)	↑FOXO3	[129]
	2.5 mg/kg/d (10 d)	Rats (heart tissue)	↑Beclin-1 ↑LC3-II	[123]
	1.8 mg/kg/d (0–120 min)	Rats (brain tissue)	↑AMPK ↑Beclin-1 ↑LC3-II	[130]
	100 mg/kg/d (14 d)	Piglets (intestinal tissue)	↑PINK1 ↑PARKIN ↑LC3-II/LC3-I	[26]
	2.5 mg/kg/d (15 d)	Rats (heart tissue)	↑PINK1 ↑PARKIN ↑SIRT1 ↑SIRT3 ↑FOXO3	[131]
	20 mg/L (1 d/10 d)	Zebrafish (retinal tissue)	↑AMPK ↑PGC-1α ↑SIRT1 ↓AKT ↓mTOR ↑Fis1 ↑PINK1	[132]
**Spermidine**				
**In vitro**	100 nM (24 h)	Chondrocytes	↑Beclin-1 ↑LC3-II	[133]
	50 μM (8 h)	GM00637 normal fibroblast cells	↑PINK1 ↑PARKIN	[134]
	5, 20, 50 μM (1 h)	N2a mouse neuroblastoma cells	↑LC3-II	[40]
	100 nM (2 h)	Isolated human chondrocytes and HTB-94 chondrosarcoma cells	↑ULK1 ↑Beclin-1 ↑Atg5 ↑Atg7 ↑LC3 ↑LC3-II	[135]
	100 µM (2 h)	HCT 116 human colon cancer cells	↑Atg5 ↑LC3	[136]
	100 µM (4 h)	HeLa human cervical cancer cells and MEF mouse embryonic fibroblasts	↑LC3-II	[137]
	10, 100, 1.000 μM (12 h)	Neonatal rat cardiomyocytes	↑AMPK ↓mTOR ↑LC3-II	[138]
	100 μM (4 h)	U2OS human bone osteosarcoma epithelial cells	↓S6K1 ↑LC3-II	[139]
**In vivo**	3 mM (4 w)	Mice (liver tissue)	↑AMPK ↑ULK1 ↓mTOR ↑Beclin-1 ↑LC3-II/LC3-I	[140]
	50 mg/kg/d (3 h)	Mice	↑LC3	[136]
	50 mg/kg/d (3 h)	Mice (hepatocytes)	↑LC3-II	[137]
	2 mM (8 w)	SAMP8 senescence-accelerated mouse-prone 8, mice model for aging (brain tissue)	↑AMPK ↑Beclin-1 ↑LC3-II	[141]
	5 mM (4 w)	Rats (cardiomyocytes)	↑AMPK ↓mTOR ↑LC3-II	[138]
	5 mg/kg/d (42 d)	Rats (skeletal muscle tissue)	↑AMPK ↑FOXO3 ↑Beclin-1 ↑LC3-II/LC3-I	[142]
	3 mM (12 w)	Mice (cardiomyocytes)	↑Atg5 ↑LC3-II	[143]
	3 mM (4 w)	Mice (thoracic aorta tissue)	↑Atg3 ↑LC3-II	[144]

Abbreviations: ↑ = upregulation; ↓ = downregulation; AKT = protein kinase B; AMPK = AMP-activated protein kinase; Atg = autophagy-related gene; BNIP3 = Bscl2 interaction protein 3; d = day; Drp1 = dynamin-related protein 1; ERK = extracellular signal-regulated kinase; EVOO = extra virgin olive oil; Fis1 = fission protein 1; FOXO 1/3 = forkhead transcription factor 1/3; FUNDC1 = FUN14 domain containing 1; h = hour; LC3-II = microtubule-associated protein 1A/1B-light chain 3; m = month; mg/kg/d = milligram/kilogram/day; Mg/L = milligram/liter; Min = minute; mM = millimol/liter; mTOR = the mammalian target of rapamycin; nM = nanomol/liter; NRF 1/2 = nuclear respiratory factor 1 and 2; PARKIN = cytosolic E_3_ ubiquitin ligase; PGC-1 α/β = peroxisome proliferator activated receptor gamma coactivator-1 alpha/beta; PINK1 = PTEN-induced kinase 1; S6K1 = ribosomal protein S6 kinase beta 1; SIRT 1/3 = silent mating type information regulation 2 homolog 1/3; TFAM = mitochondrial transcription factor A; TFEB = transcription factor EB; ULK1 = Unc-51-like kinase 1; w = weeks; μM = μmol/liter.

## Abbreviations

3xTg mice	Triple transgenic mice
AD	Alzheimer’s disease
AGS	Human gastric epithelial cells
AKT	Protein kinase B
AMBRA1	Activating molecule in Beclin 1-regulated autophagy protein 1
AMPK	AMP-activated protein kinase
Aß	Amyloid ß-peptide
Atg	Autophagy-related gene
ATP	Adenosine triphosphate
BNIP3	Bscl2 interaction protein 3
C-28/I2	Human chondrocytes
*C. elegans*	*Caenorhabditis elegans*
CREB	CAMP response element-binding protein
d	Day
db/db	Diabetic mouse model
DNA	Deoxyribonucleic acid
Drp1	Dynamin-related protein 1
EA.hy 926	Human umbilical vein endothelial cells
ER	Endoplasmic reticulum
ERK	Extracellular signal-regulated kinase
EVOO	Extra virgin olive oil
Fis1	Fission protein 1
FKBP8	FK 506 binding protein 8
FOXO 1/3	Forkhead transcription factor 1/3
FUNDC1	FUN14 domain containing 1
GM00637	Normal fibroblast cells
GTP	Guanosine triphosphate
h	Hour
H9c2	Cardiac myoblast cells
HCT 116	Human colon cancer cells
HeLa	Human cervical cancer cell line derived from Henrietta Lacks
HIF-1 α	Hypoxia-inducible factor α
HMrSV5	SV40 immortalized human peritoneal mesothelial cell line
HTB-94 cells	Chondrosarcoma cells
HUVECs	Human umbilical vein endothelial cells
IMM	Inner mitochondrial membrane
IPEC-J2	Intestinal porcine epithelial cell line-J2
IR/IGF-R	Insulin receptor/insulin-like growth factor receptor
LC3-II	Microtubule-associated protein 1A/1B-light chain 3
LIR	LC3-interacting regions
m	Month
MD	Mediterranean diet
Mdx mice	Model for studying Duchenne muscular dystrophy
MEF	Mouse embryonic fibroblast
Mfn 1/2	Mitofusin 1/2
mg/kg/d	Milligram/kilogram/day
Mg/L	Milligram/liter
Min	Minute
Mitogenesis	Mitochondrial biogenesis
mM	Millimol/liter
MMP; ΔΨm	Mitochondrial membrane potential
mtDNA	Mitochondrial DNA
mTOR	The mammalian target of rapamycin
N2a	Neuro2a mouse neuroblastoma cells
NDP52	52-kDa nuclear dot protein
NIX	Nip3-like protein X
nM	Nanomol/liter
NRF 1/2	Nuclear respiratory factor 1 and 2
OA	Osteoarthritis
OK	Okinawa
OMM	Outer mitochondrial membrane
OPA1	Optic atrophy 1
OXPHOS	Oxidative phosphorylation
p-tau	Hyperphosphorylated tau
PARKIN	Cytosolic E_3_ ubiquitin ligase
PARL	Presenilin-associated rhomboid-like
PGC-1	Peroxisome proliferator activated receptor gamma coactivator-1
PHB2	Prohibitin 2
PI3K	Phosphoinositide 3-kinases
PINK1	PTEN-induced kinase 1
PTEN	Phosphatase and tensin homolog
RNS	Reactive nitrogen species
ROS	Reactive oxygen species
RSV rats	Respiratory syncytial virus rats
S6K1	Ribosomal protein S6 kinase beta 1
SAMP8	Senescence accelerated mouse-prone 8
SH-SY5Y	Human neuroblastoma cells
SIRT 1/3	Silent mating type information regulation 2 homolog 1/3
SMURF1	SMAD ubiquitination regulatory factor 1
TCA	Tricarboxylic acid
TFAM	Mitochondrial transcription factor A
TFEB	Transcription factor EB
TgCRND8	Transgenic mouse model of AD
TIM	Translocase of the inner membrane
TOM	Translocase of the outer membrane
U-87 MG	Uppsala 87 malignant glioma cells
U2OS cells	Human bone osteosarcoma epithelial cells
U373-MG	Uppsala 373 malignant glioma cells
ULK1	Unc-51-like kinase 1
UNESCO	United Nations Educational, Scientific and Cultural Organization
USA	United States of America
VDAC	Voltage-dependent anion channel
VSMC	Vascular smooth muscle cells
w	Weeks
μM	μmol/L
ω3	Omega 3
